# Intervertebral Disc Degeneration and Regeneration: New Molecular Mechanisms and Therapeutics

**DOI:** 10.3390/cells13020153

**Published:** 2024-01-15

**Authors:** Hideki Sudo

**Affiliations:** Department of Advanced Medicine for Spine and Spinal Cord Disorders, Faculty of Medicine and Graduate School of Medicine, Hokkaido University, N15W7, Sapporo 060-8638, Hokkaido, Japan; hidekisudo@yahoo.co.jp

The intervertebral disc (IVD) is a soft tissue that constitutes the spinal column together with the vertebrae, and consists of the central nucleus pulposus (gelatinous tissue) and the annulus fibrosus (rich in fibrous tissue) that surrounds the nucleus pulposus. The regeneration capacity of IVDs is limited because viable cells are scarce and poor nutritional supply is provided to the cells of the avascular IVD [1].

The increasing incidence of IVD degeneration with age and its correlation with lower back pain, IVD herniation, and spinal canal stenosis is a remarkable trend in contemporary society [2]. Although neural decompression and/or spinal fusion are effective surgical treatments, they do not focus on the etiology of IVD degeneration, which is poorly understood. Therefore, a novel and fundamental approach to treating IVD degeneration is highly anticipated. This Special Issue aimed to report the current knowledge on the molecular mechanisms of degeneration and regeneration of the IVD for new treatment strategies. In total, six papers were finally accepted for publication and inclusion in this Special Issue (four articles and two reviews).

Song-Shu Lin’s article (contribution 1) investigated mir-107/Wnt3a-β-catenin signaling following hyperbaric oxygen (HBO) intervention using human nucleus pulposus cells. This research further discusses the protective nature of HBO against IVD degeneration in a rabbit degenerated IVD model. 

The article by Saravi et al. (contribution 2) is focused on components of the tissue renin–angiotensin system, showing the association with inflammatory and degenerative processes in human nucleus pulposus cells.

The article by Suzuki et al. (contribution 3) aimed to investigate the therapeutic efficacy of injecting a mixture of ultra-purified, Good Manufacturing Practice-compliant human bone marrow mesenchymal stem cells and alginate for discogenic pain and IVD regeneration in a rat degenerated IVD model.

From a different perspective than the above three research articles, Damle et al. (contribution 4) discusses whether IVD destruction with proteoglycanase and/or generating an IVD blood supply could make the IVD permissive to osteogenesis and fusion.

A review by Ohshini et al. (contribution 5) provides a comprehensive overview of the molecules, scaffolds, and environmental factors that facilitate the differentiation of mesenchymal stem cells into IVD cells for regenerative therapies for IVD degeneration.

Contribution 6, by Chen et al., focuses on the ferroptosis of IVD cells. Ferroptosis is characterized by iron-dependent lipid peroxidation and has been implicated in the pathological cell death associated with degenerative diseases [3]. This review article discusses its molecular pathways and biomarkers for treating IVD degeneration. 

Thus, the current articles presented in this Special Issue should be seen not only as the results of the investigations carried out by the respective researchers, but also provide readers with the current knowledge on the molecular mechanisms of degeneration and regeneration of the IVD for new treatment strategies.

## List of Contributions

1. Lin, S.-S.; Ueng, S.W.N.; Chong, K.-Y.; Chan, Y.-S.; Tsai, T.-T.; Yuan, L.-J.; Liu, S.-J.; Yang, C.-Y.; Hsiao, H.-Y.; Hsueh, Y.-J.; et al. Effects of Hyperbaric Oxygen Intervention on the Degenerated Intervertebral Disc: From Molecular Mechanisms to Animal Models. *Cells* **2023**, *12*, 2111. https://doi.org/10.3390/cells12162111.
2. Saravi, B.; Li, Z.; Basoli, V.;Grad, S.; Häckel, S.; Albers, C.E.; Alini, M.; Schmal, H.; Obid, P.; Lang, G. In Vitro Characterization of a Tissue Renin-Angiotensin System in Human Nucleus Pulposus Cells. *Cells* **2022**, *11*, 3418. https://doi.org/10.3390/cells11213418.
3. Suzuki, H.; Ura, K.; Ukeba, D.; Suyama, T.; Iwasaki, N.; Watanabe, M.; Matsuzaki, Y.; Yamada, K.; Sudo, H. Injection of Ultra-Purified Stem Cells with Sodium Alginate Reduces Discogenic Pain in a Rat Model. *Cells* **2023**, *12*, 505. https://doi.org/10.3390/cells12030505.
4. Damle, S.R.; Krzyzanowska, A.K.; Korsun, M.K.; Morse, K.W.; Gilbert, S.; Kim, H.J.; Boachie-Adjei, O.; Rawlins, B.A.; van der Meulen, M.C.H.; Greenblatt, M.B.; et al. Inducing Angiogenesis in the Nucleus Pulposus. *Cells* **2023**, *12*, 2488. https://doi.org/10.3390/cells12202488.
5. Ohnishi, T.; Homan, K.; Fukushima, A.; Ukeba, D.; Iwasaki, N.; Sudo, H. A Review: Methodologies to Promote the Differentiation of Mesenchymal Stem Cells for the Regeneration of Intervertebral Disc Cells Following Intervertebral Disc Degeneration. *Cells* **2023**, *12*, 2161. https://doi.org/10.3390/cells12172161.
6. Chen, J.; Yang, X.; Feng, Y.; Li, Q.; Ma, J.; Wang, L.; Quan, Z. Targeting Ferroptosis Holds Potential for Intervertebral Disc Degeneration Therapy. *Cells* **2022**, *11*, 3508. https://doi.org/10.3390/cells11213508.
